# HIV Knowledge and Risk Behaviors Among Older Church-Affiliated Blacks

**DOI:** 10.1177/2333721419855668

**Published:** 2019-06-27

**Authors:** Araba A. Kuofie, Alex Bauer, Jannette Berkley-Patton, Carole Bowe-Thompson

**Affiliations:** 1 University of Kansas, Lawrence, USA; 2 University of Missouri–Kansas City, USA

**Keywords:** HIV/AIDS, knowledge, risk behaviors, older Blacks

## Abstract

There is an emerging population of older adults living with HIV, and among them, Black older adults experience the greatest burden of the disease. This is a growing public health concern, as older adults are disproportionately diagnosed at a later stage of the disease, while reporting similar risk factors as younger adults. It has also been shown that the Black Church is well positioned to offer health screenings. Thus, this study aimed to assess HIV knowledge, beliefs, and risk behaviors of older church-affiliated Black adults. Data were collected from a sample of Black adults (*N* = 543) from four predominately Black churches in Kansas City, MO. Participants were surveyed on measures assessing demographic characteristics, HIV knowledge and attitudes, and HIV testing and risk behaviors. Results indicated that compared to younger Black adults, Black older adults were less knowledgeable about the transmission of HIV and were less willing to be tested for HIV in church settings. However, there was no significant difference on the perceived seriousness of HIV in the community. Results further showed that Black older adults were less likely to use condoms/barriers during the past 6 months and over their lifetime. We discuss the implications of results for HIV intervention programs.

While young adults account for most HIV cases, there is an emerging population of older adults—individuals aged 50 and older (Centers for Disease Control and Prevention, 2017b)—living with HIV. Among older adults with HIV, Blacks—identifying as American, Caribbean or West Indian, African, or another non-American descent (Griffith, Johnson, Zhang, Neighbors, & Jackson, 2011) experience the greatest burden of this disease. In 2015, Blacks accounted for an estimated 43% of all new HIV diagnoses among people aged 50 and above (Centers for Disease Control and Prevention, 2017b).

In general, Blacks are at a greater risk for experiencing certain socioeconomic constraints (e.g., inadequate health care, poverty, lack of education, stigma, and discrimination), which places them at an increased risk for HIV infection (Centers for Disease Control and Prevention, 2017a; Morbidity and Mortality Weekly Report, 2013). For example, stigma and discrimination may prevent Black older adults from seeking HIV care and/or disclosing their HIV status, thereby increasing their risks for transmission (Centers for Disease Control and Prevention, 2017a). In addition to these constraints, Black older adult**s** also face unique challenges that occur as a result of aging (e.g., isolation due to illness, loss of family and friends, poorer quality of life, failure to distinguish HIV symptoms from those of normal aging, low perceived risk for HIV, and decreased likelihood of being screened by providers), which may increase their risk for HIV (Centers for Disease Control and Prevention, 2017b).

Although at a disproportionate risk for HIV infection, Black older adults have been understudied in this area of research. However, existing research suggests that Black older adults still hold several misconceptions regarding the transmission of the disease, such as the beliefs that HIV can be acquired through casual contact (e.g., sharing a toilet, sharing drinks, and shaking hands), and avoiding sex with individuals who are dirty eliminates your risk of HIV infection (Emlet et al., 2015; Sankar, Nevedal, Neufeld, Berry, & Luborsky, M., 2011; Ward, Disch, Schensul, & Levy, 2011). While Black older adults generally regard HIV as a serious disease, many do not believe they are susceptible to acquiring HIV (Adekeye, Heiman, Onyeabor, & Hyacinth, 2012; Sankar et al., 2011). Unaware of their risks, many Black older adults engage in risk behaviors that increase their susceptibility to the disease. For instance, existing research suggests that many Black older adults engage in sex with multiple partners and are reluctant to use condoms during sexual activities (Bowdre, 2013; Hawes & Berkley-Patton, 2014; Sankar et al., 2011). These findings present a public health concern, and as a result, increased attention is now being given to Black older adults.

## The Black Church

A 2014 national survey revealed that approximately 82% of Black adults reported a religious affiliation. Furthermore, 86% of Black adults reported praying daily or weekly, and 83% reported regular attendance at religious services (Pew Research Center: Religion & Public Life, 2014). Moreover, it has been established that Black older adults have higher religious involvement (e.g., regular church attendance and engagement in church activities) than younger Black adults and other racial and ethnic groups (Brown, Taylor, & Chatters, 2015).

Predominately Black churches represent a safe haven for the Black community and are often fundamental to the lives, beliefs, and behaviors of many Blacks (Wortham, 2009). As one of the few institutions left relatively free by White society in its development, the Black church is a trusted entity by members of the Black community (Nelsen & Nelsen, 2015). During the period of slavery, the church provided solace to Blacks escaping persecution and harassment (Mellowes, 2010). It was a place where slaves gathered and engaged in worship that emphasized deliverance from the troubles of the present world. In the times of the civil war, the church was instrumental in the abolition of slavery (Mellowes, 2010). The Black church continues to provide social solidarity to the community, often offering a wide range of social and health services while also advocating for social justice (Nelsen & Nelsen, 2015). Therefore, the Black church has the potential to serve as a venue for health promotion and educational programs focusing on conditions such as HIV.

## Aims

Few studies have examined HIV knowledge and behaviors among older adults, and even fewer have specifically focused on Black older adults. The purpose of this study was to investigate HIV knowledge, beliefs, testing behaviors, and the prevalence of HIV risk behaviors (i.e., multiple sex partners and inconsistent condom use) among older, church-affiliated, Black adults.

## Methods

### Contextual Background

This study was conducted as part of Project Taking It to the Pews (TIPS), a church-based intervention designed to promote HIV awareness and HIV testing in four urban Black churches in Kansas City, Missouri, and Kansas. Guided by a faith community-engaged approach, TIPS was designed for delivery through multilevel church outlets (e.g., peer-to-peer, ministry groups, church services, and community outreach services) by trained church leaders with the support of the TIPS Awareness, Prevention, and Compassion Tool Kit, packaged with religiously tailored materials to promote HIV testing (e.g., sermon guides, testimonials, responsive readings, church bulletin inserts, and bible bookmarks; Berkley-Patton et al., 2012). The primary outcome was receipt of HIV testing. Church-affiliated participants (i.e., church and community members) completed a baseline survey as part of the TIPS pilot study (see Berkley-Patton et al., 2018, for complete description).

### Participants and Procedures

A total of 543 participants from four Black churches and their community outreach programs (e.g., food/clothing pantry, recovery programs, and social services) were included in the study. Participants completed a survey on health beliefs and behaviors. Among participants, 121 (47 men and 74 women; mean age = 59.42 years, *SD* = 2.93 years) were categorized as older adults (aged 55+ years). Surveys were completed at the respective churches and church outreach programs of all participants. Each participant provided informed consent and received a $10 cash reimbursement for survey completion. Surveys took 20 to 30 minutes to complete. This study was approved by the University of Missouri—Kansas City Institutional Review Board.

### Survey Measures

#### Participant characteristics

Demographics were collected to describe the participants (Table 1). Participants were asked to write in their age and report their gender (i.e., male or female). Participants were also asked to report their highest level of education (ranging from 1 = *sixth grade or less* to 8 = *some graduate school or graduate degree*), marital status (i.e., single, never married; living with partner, but not married; married; separated; divorced; and widowed), and average monthly household income (ranging from $0-$1000 to greater than $3000).

**Table 1.** Participant Characteristics.

	Younger adults (*N* = 422) % (*n*)	Older adults (*N* = 121) % (*n*)	Overall (*N* = 543) % (*n*)	*χ*^2^ (*p*)
Gender				5.09 (.475)
Male	35.3 (149)	38.8 (47)	36 (196)	
Female	64.7 (273)	61.2 (74)	64 (347)	
Marital Status				67.01 (<.001)
Single/never married	44.9 (189)	15.0 (18)	38.3 (207)	
Cohabitating, not married	6.9 (29)	1.7 (2)	5.7 (31)	
Married	31.1 (131)	37.5 (45)	32.5 (176)	
Separated	4.0 (17)	8.3 (10)	5.0 (27)	
Divorced	11.6 (49)	27.5 (33)	15.2 (82)	
Widowed	1.4 (6)	10 (12)	3.3 (18)	
Education level				8.16 (.319)
7th-11th grade	8.3 (35)	4.2 (5)	7.4 (40)	
High school/GED	23.7 (100)	31.7 (38)	25.5 (138)	
Technical training	1.9 (8)	3.3 (4)	2.2 (12)	
Some college	28.7 (121)	26.7 (32)	28.2 (153)	
Associate’s degree	11.8 (50)	7.5 (9)	10.9 (59)	
Bachelor’s degree	11.4 (48)	14.2 (17)	12.0 (65)	
Some graduate/graduate	14.0 (59)	12.5 (15)	13.7 (74)	
Avg. monthly income				5.99 (.307)
$0-$1,000	18.6 (78)	18.5 (22)	18.6 (100)	
$1,001-$2,000	16.7 (70)	14.3 (17)	16.1 (87)	
$2,001-$2,500	9.3 (39)	9.2 (11)	9.3 (50)	
$2,501-$3,000	11.4 (48)	11.8 (14)	11.5 (62)	
>$3,000	31.7 (133)	40.3 (48)	33.6 (181)	

*Note.* Percentages are based on valid percent. GED = General Educational Development.

#### Receipt of HIV testing

Participants’ self-reported receipt of HIV testing was measured with one dichotomous item (*Yes* = 1, *No* = 0). This item was measured using an item adapted from national surveys, and our pilot studies were conducted among older adults (55 and older) and African American populations (e.g., Berkley-Patton et al., 2013; Centers for Disease Control and Prevention, 2009).

#### HIV sexual risk behaviors

Participants reported their number of sex partners (lifetime and past 6 months) and how consistently they used condoms (lifetime and past 6 months; *Always* [100%] = 0 to *Never* [0%] = 3; Table 2) (Hawes et al., 2014).

**Table 2.** HIV Risk Behaviors.

	Younger adults *M* (*SD*)	Older adults *M* (*SD*)	Overall *M* (*SD*)	*p*
Sex partners				
Past 6 months	1.44 (1.24)	1.10 (.67)	1.39 (1.18)	.045*
Lifetime	13.68 (24.75)	11.49 (15.98)	13.24 (23.26)	.434
Condom use				
Past 6 months	2.05 (1.15)	2.48 (.97)	2.11 (1.13)	.009*
Lifetime	1.56 (.92)	2.04 (.93)	1.66 (.94)	.000*

* *p* < .05.

#### Willingness to be tested for HIV in church settings

Participants were asked, “*If HIV testing was available at your church/church-sponsored event, would you take advantage of this HIV testing?*” with response options ranging from *Definitely no* = 0 to *Definitely yes* = 4.

#### Perceived seriousness of HIV

Participants were asked, “*How serious of a health issue is HIV in your community?*” with response options ranging from *Not at all serious* = 0 to *Very Serious* = 4.

#### HIV knowledge

Ten items from the HIV Knowledge Questionnaire (HIV-K-Q; Carey, Morrison-Beedy, & Johnson, 1997) were used to assess HIV knowledge (e.g., “*You can get HIV if you share a drink, sink, shower, or toilet seat with someone who has AIDS*”; “*A pregnant woman who has HIV can give it to her baby, before, during and after childbirth*”). Participants were asked to check “True,” “False,” or “Don’t know.” False items were reverse-scored, and items were summed to create a single HIV knowledge score ranging from 0 (*low knowledge*) to 10 (*high knowledge*). The HIV-K-Q has been validated among older adults (55 and older) and African American populations and found to be reliable with an internal consistency of .91 (Cronbach’s α; Carey et al., 1997).

### Data Analyses

Data were analyzed using the Statistical Package for Social Sciences software (SPSS), Version 24 (IBM Corp., 2016). Descriptive statistics and chi-square (*χ*^2^) analyses were used to assess differences in participant characteristics. One-way analyses of variance (ANOVAs) were performed to examine mean differences between older adults (i.e., participants aged 55 and older) and younger adults for total HIV knowledge scores, receipt of HIV testing (i.e., yes/no), perceived seriousness of HIV, number of sexual partners, condom use, and willingness to test for HIV in church settings.

## Results

Overall, participants were primarily female (64%, *n* = 347), with an average age of 42.3 years (*SD* = 13.5). Most of the younger Black adults reported being single/never married (45%, *n* = 189), a monthly income of less than $3,000 (56%, *n* = 235), and a high-school degree or beyond (91.5, *n* = 386). Among Black older adults, most reported being married (38%, *n* = 45) or divorced (28%, *n* = 33), a monthly income of less than $3,000 (53.8%, *n* = 64), and a high-school degree or beyond (95.9%, *n* = 115).

Results indicated that a slight majority of Black older adults (56%, *n* = 66) had been tested for HIV in their lifetime. Black older adults (aged 55 and above) were significantly less likely to report receipt of testing compared to participants aged 54 and younger (*F* [1,535] = 35.36, *p* < .001). Black older adults were also less likely to report using condoms/barriers in the past 6 months (*F* [1,382] = 7.00, *p* < .009) and over their lifetime (*F* [1,476] = 21.22, *p* < .000). Younger Black adults were more likely to report vaginal, anal, and/or oral sex within the past 6 months (*F* [1,455] = 55.45, *p* < .000) and in their lifetime (*F* [1,447] = 10.89, *p* < .001). Although younger Black adults reported a greater number of sex partners in the past 6 months (*F* [1,386] = 4.03, *p* < .045), there was no significant difference in lifetime sex partners among the two groups.

Compared to younger Black adults, Black older adults also showed less knowledge about the transmission of HIV, *t* (512) = 2.02, *p* = .044, and were less willing to be tested for HIV in church settings (*t* [175] = 2.58, *p* = .011). There was no significant difference in the perceived seriousness of HIV in the community between the two groups, *t* (525) = –.775, *p* = .439. Most Black younger (76%) and older adults (75%) similarly perceived HIV to be a very serious health condition.

## Discussion

This study investigated HIV knowledge, beliefs, testing behaviors, and the prevalence of HIV risk behaviors of older church-affiliated Black adults. Our findings indicated that Black older adults were less likely to report receipt of HIV testing, had less knowledge about the transmission of HIV, and were less willing to be tested for HIV in church settings than their younger counterparts. Although other studies have investigated HIV knowledge and behaviors among Black church-goers, very few have examined the willingness of this population to address HIV-related issues in the church setting.

Research indicates that Black older adults have higher religious involvement than younger Black adults (Brown et al., 2015). Moreover, the Black church has played a role in the delivery and implementation of health-promotion interventions focused on various health concerns including obesity and weight loss (Lancaster, Carter-Edwards, Grilo, Shen, & Schoenthaler, 2014; Wasserman, Reider, & Brown, 2010), physical activity (Whitt-Glover, Hogan, Lang, & Heil, 2008; Wilcox et al., 2007), substance use (Stahler, Kirby, & Kerwin, 2007), diabetes prevention (Boltri et al., 2008), and cancer screenings (Drake, Shelton, Gilligan, & Allen, 2010; Matthews, Berrios, Darnell, & Calhoun, 2006). However, Black older adults in this study were less willing than younger Black adults to seek out HIV testing in church settings. A simple explanation for this may be the common assumption that older adults tend to be more conservative in their beliefs and attitudes. Therefore, discussions of sensitive health-related topics (e.g., HIV and sexual behavior) in such a conservative institution may be less welcomed by Black older adults.

Several strategies have been proposed to promote church-based HIV testing among Black older adults. Conceivably, more discussions in church settings about human sexuality and the risks of contracting HIV may encourage older church members to be more receptive toward church-based interventions. Since evidence shows that church leaders are willing to discuss HIV-related issues with the community (Francis, Lam, Cance, & Hogan, 2009; Nunn et al., 2012), they could take the lead and begin by confronting the issue of HIV-related stigma. In addition, church leaders can organize health ministries or committees that serve the purpose of discussing HIV and health-related issues (Coleman, Lindley, Annang, Saunders, & Gaddist, 2012). This would create a norm of discussing sensitive health topics within the church.

Furthermore, the recruitment of older church members in the development and execution of intervention programs may also increase the willingness of individuals from that population to participate (Coleman et al., 2012). Essentially, if members can identify with those in charge of the program, they may be more likely to “buy-into” the intervention. Alternatively, HIV intervention programs could use other venues that specifically serve Black older adults. For example, community centers, senior centers, and Black businesses such as barbershops and salons may offer a less conservative setting to discuss sensitive health issues (Lindau, Leitsch, Lundberg, & Jerome, 2006). Ultimately, further research is warranted to identify the specific reasons some Black older adults have for their apprehension toward receiving HIV testing in church settings. Findings from future studies could result in modifications for church-based HIV interventions among Black older adults.

Findings from this study showed that Black older adults engaged in sex with multiple partners and were less likely than younger Black adults to use condoms or barriers. This is concerning because unsafe sexual practices such as having multiple sexual partners and having unprotected sex are associated with an increased risk of HIV infection (Centers for Disease Control and Prevention, 2015). Although these findings are consistent with previous literature, this study extends this pattern of risk behavior to older church-affiliated Black adults. It has been suggested that older adults are reluctant to use condoms/barriers during sex because they consider themselves to be beyond childbearing years, and thus see no need to use protection (Lindau et al., 2006). This belief is likely prevalent among Black older adults, given the findings of this study and should be an area of concern for interventions targeting this group. Educational interventions should address this belief to increase awareness of personal susceptibility to HIV among older adults. In addition, further research could reveal other reasons for the resistance to condom use among Black older adults to better inform intervention programs.

Although Black older adults in this study engaged in similar risk behaviors as younger Black adults, results showed that they were not getting tested as recommended. This finding is also consistent with existing research that suggests that older adults are less likely to be tested for HIV (Ford et al., 2015; Schick et al., 2010). Black older adults may be reluctant to seek out HIV testing for several reasons. First, they may be apprehensive about getting tested due to stigma (Centers for Disease Control and Prevention, 2017a). Second, they may be diagnosed with age-related diseases, which can make it difficult to detect changes in health related to HIV. Moreover, if signs of HIV are mistaken for normal aging, it can lead to delayed testing and treatment seeking (Centers for Disease Control and Prevention, 2017b). Third, it has been reported that older adults are disproportionately diagnosed in later stages of HIV (Centers for Disease Control and Prevention, 2017b); hence, it may be too late to seek care by the time they are diagnosed. Finally, due to incorrect assumptions about the sexual lives of older adults, health-care providers may not recommend testing for Black older patients because they may perceive them to be at low risk for sexually transmitted diseases (Centers for Disease Control and Prevention, 2017b; Emlet, 2006; Ford et al., 2015; Tillman & Mark, 2015).

Thus, the stereotype that older adults are sexually inactive leaves them ignored when it comes to sexual health screening and education (Pilowsky & Wu, 2015). Once again, this is where partnering with the Black church and church leaders may be beneficial. Specifically, church leaders can serve an important role in raising awareness about health screenings. These influential leaders also can encourage participation and maintenance in health intervention programs (i.e., participation in health screenings and upkeep of skills acquired from the interventional program). Furthermore, church-based programs are generally cost-effective for those who participate because many of these programs are free and/or provide an incentive for participation (Williams et al., 2013). As a result, uninsured or low-income members of the community tend to find these programs appealing (Williams et al., 2013). More importantly, the Black church provides a way to culturally tailor interventions (e.g., by integrating the structural elements of the community, such as values and beliefs, into the programs) to make them more suitable for the community.

Finally, results also showed that there was no significant difference in the perceived seriousness of HIV between Black younger and older adults. Black younger and older adults in this study both perceived HIV to be a serious health condition but still engaged in several risk behaviors. Two important conclusions arise from these findings. First, education regarding the seriousness of HIV may mitigate but not eliminate an individual’s engagement in HIV risk behaviors. Second, educational interventions for Black older adults will need to emphasize in their curriculum, HIV modes of transmission, and personal susceptibility to the disease.

### Limitations

While this study adds to the limited information available on HIV knowledge, beliefs, and risk behaviors among Black church-affiliated populations, study limitations still exist. As the study did not examine differences between church and community members, it is difficult to conclude whether this characteristic influenced the findings. In addition, this study did not specifically assess perceived risk for HIV infection; therefore, it may be hard to draw conclusions about personal vulnerability to HIV. Finally, given the study’s reliance on a self-reported questionnaire, poor recall, or socially desirable reporting of HIV risk behaviors may have occurred, meaning that HIV risk behaviors may be underrepresented. If this happened, participants may be at even greater risk for HIV, thereby further signifying the critical need for more HIV interventions for this population.

### Conclusion

Among older adults living with HIV, Blacks are disproportionately impacted. They report similar risk factors as younger Black adults but experience lower testing rates. Despite the positive impact of religiosity on health outcomes among the Black population, older church-affiliated Black adults still engage in behaviors that put them at risk for HIV. In addition, church-based interventions may miss opportunities to address issues specifically related to older church-affiliated Black adults. To that end, further investigation is required to address Black older adults’ apprehension toward receiving HIV testing in church settings. With Black older adults disproportionately burdened with HIV, it is imperative for HIV intervention programs to be culturally sensitive to the needs of this population, while providing HIV education that highlights personal susceptibility to the disease.

## References

[bibr1-2333721419855668] Adekeye O. A. Heiman H. J. Onyeabor O. S. Hyacinth H. I. (2012). The new invincibles: HIV screening among older adults in the US. PLoS ONE, 7(8), e43618. 22952722 10.1371/journal.pone.0043618 PMC3428311

[bibr2-2333721419855668] Berkley-Patton J. Bowe Thompson C. Moore E. Hawes S. Berman M. Allsworth A. Goggin K. (2018). Feasibility and outcomes of an HIV testing intervention in African American churches. AIDS and Behavior. 10.1007/s10461-018-2240-0 30121728

[bibr3-2333721419855668] Berkley-Patton J. Martinez D. Bowe-Thompson C. Hawes S. Moore E. Williams E. Wainright S. (2012). Examining church capacity to develop and disseminate a religiously-appropriate HIV Tool Kit with African American churches. Journal of Urban Health. doi:10.1007/s11524-012-9740-4 PMC3665971 22815053

[bibr4-2333721419855668] Berkley-Patton J. Y. Moore E. Berman M. Simon S. D. Thompson C. B. Schleicher T. Hawes S. M. (2013). Assessment of HIV-related stigma in a U.S. faith-based HIV education and testing intervention. Journal of the International AIDS Society, 16, Article 18644. 10.7448/IAS.16.3.18644 PMC3833192 24242259

[bibr5-2333721419855668] Boltri J. M. Davis-Smith Y. M. Seale J. P. Shellenberger S. Okosun I. S. Cornelius M. E. (2008). Diabetes prevention in a faith-based setting: Results of translational research. Journal of Public Health Management and Practice, 14, 29-32. 18091037 10.1097/01.PHH.0000303410.66485.91

[bibr6-2333721419855668] Bowdre T. L. (2014). Hiv general knowledge and risk behaviors in the african american older adult population (Order No. AAI3595796). Available from PsycINFO. (1554230914; 2014-99140-407). Retrieved from http://www2.lib.ku.edu/login?url=https://search-proquest-com.www2.lib.ku.edu/docview/1554230914?accountid=14556

[bibr7-2333721419855668] Brown R. K. Taylor R. J. Chatters L. M. (2015). Race/ethnic and social-demographic correlates of religious non-involvement in America: Findings from three national surveys. Journal of Black Studies, 46, 335-362.

[bibr8-2333721419855668] Carey M. P. Morrison-Beedy D. Johnson B. T. (1997). HIV Knowledge Questionnaire: Development and evaluation of a reliable, valid, and practical self-administered questionnaire. AIDS and Behavior, 1, 61-74.

[bibr9-2333721419855668] Centers for Disease Control and Prevention. (2009). HIV surveillance report. Retrieved from https://www.cdc.gov/hiv/library/reports/hiv-surveillance-archive.html

[bibr10-2333721419855668] Centers for Disease Control and Prevention. (2015). HIV risk behaviors. Retrieved from https://www.cdc.gov/hiv/risk/estimates/riskbehaviors.html

[bibr11-2333721419855668] Centers for Disease Control and Prevention. (2017a). HIV among African Americans. Retrieved from https://www.cdc.gov/hiv/group/racialethnic/africanamericans/index.html

[bibr12-2333721419855668] Centers for Disease Control and Prevention. (2017b). HIV among people aged 50 and over. Retrieved from https://www.cdc.gov/hiv/group/age/olderamericans/index.html

[bibr13-2333721419855668] Coleman J. D. Lindley L. L. Annang L. Saunders R. P. Gaddist B. (2012). Development of a framework for HIV/AIDS prevention programs in African American churches. AIDS Patient Care and STDs, 26, 116-124. 22149766 10.1089/apc.2011.0163

[bibr14-2333721419855668] Drake B. F. Shelton R. Gilligan T. Allen J. D. (2010). A church-based intervention to promote informed decision-making for prostate cancer screening among African-American men. Journal of the National Medical Association, 102, 164-171. 20355345 10.1016/s0027-9684(15)30521-6 PMC2913684

[bibr15-2333721419855668] Emlet C. A. (2006). “You’re awfully old to have this disease”: Experiences of stigma and ageism in adults 50 years and older living with HIV/AIDS. Gerontologist, 46, 781-790. 17169933 10.1093/geront/46.6.781

[bibr16-2333721419855668] Emlet C. A. Brennan D. J. Brennenstuhl S. Rueda S. Hart T. A. Rourke S. B. (2015). The impact of HIV-related stigma on older and younger adults living with HIV disease: Does age matter? AIDS Care, 27, 520-528. 25397643 10.1080/09540121.2014.978734

[bibr17-2333721419855668] Ford C. L. Lee S. J. Wallace S. P. Nakazono T. Newman P. A. Cunningham W. E. (2015). HIV testing among clients in high HIV prevalence venues: Disparities between older and younger. AIDS Care, 27, 189-197. 25303208 10.1080/09540121.2014.963008 PMC4286518

[bibr18-2333721419855668] Francis S. A. Lam W. K. Cance J. D. Hogan V. K. (2009). What’s the 411? Assessing the feasibility of providing African American adolescents with HIV/AIDS prevention education in a faith-based setting. Journal of Religion and Health, 48, 164-177. 19421867 10.1007/s10943-008-9177-y

[bibr19-2333721419855668] Griffith D. M. Johnson J. L. Zhang R. Neighbors H. W. Jackson J. S. (2011). Ethnicity, nativity, and the health of American Blacks. Journal of Health Care for the Poor and Underserved, 22, 142-156. 21317512 10.1353/hpu.2011.0011 PMC3326411

[bibr20-2333721419855668] Hawes S. M. Berkley-Patton J. Y. (2014). Religiosity and risky sexual behaviors among an African American church-based population. Journal of Religion and Health, 53, 469-482. 23054481 10.1007/s10943-012-9651-4 PMC3985388

[bibr21-2333721419855668] IBM Corp. (2016). IBM SPSS Statistics for Windows, Version 24.0. Armonk, NY: Author.

[bibr22-2333721419855668] Lancaster K. J. Carter-Edwards L. Grilo S. Shen C. Schoenthaler A. M. (2014). Obesity interventions in African American faith-based organizations: A systematic review. Obesity Reviews, 15, 159-176. 10.1111/obr.12207 25196412

[bibr23-2333721419855668] Lindau S. T. Leitsch S. A. Lundberg K. L. Jerome J. (2006). Older women’s attitudes, behavior, and communication about sex and HIV: A community-based study. Journal of Women’s Health, 15, 747-753. 10.1089/jwh.2006.15.747 16910906

[bibr24-2333721419855668] Matthews A. K. Berrios N. Darnell J. S. Calhoun E. (2006). A qualitative evaluation of a faith-based breast and cervical cancer screening intervention for African American women. Health Education & Behavior, 33, 643-663. 16861590 10.1177/1090198106288498

[bibr25-2333721419855668] Mellowes M. (2010). The Black church. Retrieved from http://www.pbs.org/godinamerica/black-church/

[bibr26-2333721419855668] Morbidity and Mortality Weekly Report. (2013). CDC health disparities and inequalities report—United States, 2013 (Social determinants of health, Vol. 62). Retrieved from https://www.cdc.gov/mmwr/pdf/other/su6203.pdf

[bibr27-2333721419855668] Nelsen H. M. Nelsen A. K. (2015). Black church in the sixties. Lexington: The University Press of Kentucky.

[bibr28-2333721419855668] Nunn A. Cornwall A. Chute N. Sanders J. Thomas G. James G. Flanigan T. (2012). Keeping the faith: African American faith leaders’ perspectives and recommendations for reducing racial disparities in HIV/AIDS infection. PloS one, 7(5), e36172. 10.1371/journal.pone.0036172 PMC3353968 22615756

[bibr29-2333721419855668] Pew Research Center: Religion Public Life. (2014). U.S. Religious landscape study [Data file]. Retrieved from http://www.pewforum.org/dataset/pew-research-center-2014-u-s-religious-landscape-study/

[bibr30-2333721419855668] Pilowsky D. J. Wu L. T. (2015). Sexual risk behaviors and HIV risk among Americans aged 50 years or older: A review. Substance Abuse and Rehabilitation, 6, 51-60. 25960684 10.2147/SAR.S78808 PMC4410899

[bibr31-2333721419855668] Sankar A. Nevedal A. Neufeld S. Berry R. Luborsky M. (2011). What do we know about older adults and HIV? a review of social and behavioral literature. AIDS Care, 23, 1187-1207. Retrieved from http://www2.lib.ku.edu/login?url=https://search-proquest-com.www2.lib.ku.edu/docview/897905554?accountid=14556 21939401 10.1080/09540121.2011.564115 PMC3199226

[bibr32-2333721419855668] Schick V. Herbenick D. Reece M. Sanders S. A. Dodge B. Middlestadt S. E. Fortenberry J. D. (2010). Sexual behaviors, condom use, and sexual health of Americans over 50: Implications for sexual health promotion for older adults. The Journal of Sexual Medicine, 7, 315-329. 21029388 10.1111/j.1743-6109.2010.02013.x

[bibr33-2333721419855668] Stahler G. J. Kirby K. C. Kerwin M. E. (2007). A faith-based intervention for cocaine-dependent Black women. Journal of Psychoactive Drugs, 39, 183-190. 17703713 10.1080/02791072.2007.10399877

[bibr34-2333721419855668] Tillman J. L. Mark H. D. (2015). HIV and STI testing in older adults: An integrative review. Journal of Clinical Nursing, 24, 2074-2095. 25728018 10.1111/jocn.12797

[bibr35-2333721419855668] Ward E. G. Disch W. B. Schensul J. J. Levy J. A. (2011). Understanding low-income, minority older adult self-perceptions of HIV risk. Journal of the Association of Nurses in AIDS Care, 22, 26-37. 20580270 10.1016/j.jana.2010.05.002 PMC2948611

[bibr36-2333721419855668] Wasserman T. Reider L. R. Brown B. (2010). Designing and pilot-testing a church-based community program to reduce obesity among African Americans. ABNF Journal, 21, 4-10. 20169806

[bibr37-2333721419855668] Whitt-Glover M. C. Hogan P. E. Lang W. Heil D. P. (2008). Pilot study of a faith-based physical activity program among sedentary Blacks. Preventing Chronic Disease, 5(2), A51. 18341786 PMC2396995

[bibr38-2333721419855668] Wilcox S. Laken M. Anderson T. Bopp M. Bryant D. Carter R.. . . Parrott A. W. (2007). The health-e-AME faith-based physical activity initiative: Description and baseline findings. Health Promotion Practice, 8, 69-78. 16885511 10.1177/1524839905278902

[bibr39-2333721419855668] Williams L. B. Sattin R. W. Dias J. Garvin J. T. Marion L. Joshua T.. . . Narayan K. V. (2013). Design of a cluster-randomized controlled trial of a diabetes prevention program within African–American churches: The Fit Body and Soul study. Contemporary Clinical Trials, 34, 336-347. 23354313 10.1016/j.cct.2013.01.002 PMC3594654

[bibr40-2333721419855668] Wortham R. A. (2009). W. E. B. Du Bois, the Black church, and the sociological study of religion. Sociological Spectrum, 29, 144-172. doi:10.1080/02732170802581357

